# Wettability and Contact Time on a Biomimetic Superhydrophobic Surface

**DOI:** 10.3390/ma10030254

**Published:** 2017-03-02

**Authors:** Yunhong Liang, Jian Peng, Xiujuan Li, Jubin Huang, Rongxian Qiu, Zhihui Zhang, Luquan Ren

**Affiliations:** 1 Key Laboratory of Bionic Engineering (Ministry of Education, China), Jilin University, Changchun 130025, China; liangyunhong@jlu.edu.cn (Y.L.); pengjian15@mails.jlu.edu.cn (J.P.); huangjb15@mails.jlu.edu.cn (J.H.); zhzh@jlu.edu.cn (Z.Z.); lqren@jlu.edu.cn (L.R.); 2 State Key Laboratory of Automotive Simulation and Control, Jilin University, Changchun 130025, China; 3 School of Mechatronical Engineering, Changchun University of Science and Technology, Changchun 130022, China; 15504404628@163.com

**Keywords:** wettability, array microstructure, apparent contact time, superhydrophobic, biomimetic

## Abstract

Inspired by the array microstructure of natural superhydrophobic surfaces (lotus leaf and cicada wing), an array microstructure was successfully constructed by high speed wire electrical discharge machining (HS-WEDM) on the surfaces of a 7075 aluminum alloy without any chemical treatment. The artificial surfaces had a high apparent contact angle of 153° ± 1° with a contact angle hysteresis less than 5° and showed a good superhydrophobic property. Wettability, contact time, and the corresponding superhydrophobic mechanism of artificial superhydrophobic surface were investigated. The results indicated that the micro-scale array microstructure was an important factor for the superhydrophobic surface, while different array microstructures exhibited different effects on the wettability and contact time of the artificial superhydrophobic surface. The length (*L*), interval (*S*), and height (*H*) of the array microstructure are the main influential factors on the wettability and contact time. The order of importance of these factors is *H* > *S* > *L* for increasing the apparent contact angle and reducing the contact time. The method, using HS-WEDM to fabricate superhydrophobic surface, is simple, low-cost, and environmentally friendly and can easily control the wettability and contact time on the artificial surfaces by changing the array microstructure.

## 1. Introduction

High apparent contact angles (APCAs), low contact angle hysteresis, and the highest possible stability of the Cassie wetting state are three important criteria for true superhydrophobicity [1,2,3,4,5]. The superhydrophobic property of a surface is mainly determined by the cooperation of the geometric microstructure and the low energy surface [6,7,8]. Superhydrophobic objects, such as lotus leaf, taro leaf, and India canna [9], are widespread in nature. Meanwhile, superhydrophobic surfaces have numerous promising properties, such as non-wetting [10], self-cleaning [11], anti-icing [12], lubrication [13], condensation management [14], and small flow resistance [15]. In view of so many good qualities of superhydrophobic surfaces, it is of great industrial value to fabricate functional superhydrophobic surfaces. Various methods have been developed to obtain artificial superhydrophobic surfaces, including electrospinning [16], self-assembled monolayers (SAMs) [17], the sol-gel method [18], chemical vapor deposition [19], and the template technique [20]. However, some of the above methods need severe conditions or fabricate superhydrophobic surfaces on soft materials such as polymers [21,22] and colloidal materials [23,24], which limit the application of superhydrophobic surfaces. Although methods of artificial superhydrophobic surfaces have achieved further development, several researchers have successfully fabricated superhydrophobic surfaces with specific metallic substrates (hard materials) recently, such as aluminum, steel, copper, and so on. A mechanically robust superamphiphobic aluminum surface was manufactured by simple chemical etching and anodization [7]. A robust fabrication of fluorine-free superhydrophobic steel mesh for efficient oil/water separation was performed [25]. Copper-based super-hydrophobic surface was made by oxidation, lauryl mercaptan modification, and compression molding [26]. Moreover, most of the researchers focused on the wettability of surface or the methods of manufacturing superhydrophobic surfaces, paying little attention to the dynamic contact process of droplet on the superhydrophobic surfaces. Bird has done some research on reducing the contact time of a bouncing drop on a superhydrophobic silicon surface [27]. However, silicon surfaces are soft and have limited use in engineering. If we can reduce the bouncing time (important parameter in anti-icing) on superhydrophobic aluminum alloy surfaces, the scope of superhydrophobic surfaces will be expanded in engineering applications. Therefore, it is of great necessity to pursue further analysis of reducing the contact time on the superhydrophobic aluminum alloy surfaces.

Natural superhydrophobic plant leaves greatly inspire us. Fascinated by the excellent superhydrophobic property of plant leaves, such as lotus (*Nelumbonucifera*) leaf and cicada (*Cryptotympana atrata Fabricius*) wing, and inspired by the array microstructure existing on the surfaces of lotus leaf and cicada wing, we engaged in bionic design and preparation. In this study, biomimetic superhydrophobic surfaces were successfully fabricated by high speed wire electrical discharge machining (HS-WEDM) on a 7075 aluminum alloy without any chemical modification. Meanwhile, wettability, contact time, and the corresponding superhydrophobic mechanism of an artificial superhydrophobic surface were investigated. The orthogonal experimental design on different artificial superhydrophobic surfaces was achieved to help in better understanding the wettability and contact time, and how to reduce the contact time by altering the array microstructure on the artificial surfaces. It is possible to control the wettability and the droplet contact time by changing the array microstructure on the artificial surfaces, which would have a potential application in the surface processing and preparation in anti-icing and self-cleaning.

## 2. Materials and Methods

### 2.1. Preparation of Biological Sample

The lotus leaves and cicada wings were collected from the South Lake Park of Changchun, Jilin province. The middle parts of the as-prepared cicada wings and lotus leaves were cut into about 10 × 10 mm^2^, and were rinsed using deionized water to remove environmental contaminants and then dried at room temperature. In order to measure the apparent contact angles and the contact angle hysteresis, the prepared fresh samples were adhered on glass slides using double-sided tape, ensuring each leaf as flat as possible.

### 2.2. Fabrication of Superhydrophobic Surfaces

A 7075 aluminum alloy was used as the substrate to fabricate an artificial superhydrophobic surface (length: 25 mm, width: 25 mm, thickness: 4 mm). The chemical compositions of the 7075 aluminum alloy are displayed in Table 1. The specimens were performed using HS-WEDM (DK7732, BAOMA, Suzhou, China, Working Travel: 320 × 400 mm^2^, Max Cut Taper: 60°). Molybdenum wire of 180 μm in diameter was employed as wire material (electrode). In order to avoid the phenomenon of wire breaking in the machining process, an aqueous solution of non-toxic line cut emulsion (JR3A emulsion solution) was used to improve the level of the processing technology. Machining parameters of HS-WEDM: pulse width is 16 μs, pulse interval is 112 μs, power tube number is 3, and wire speed is 50 Hz. The artificial superhydrophobic specimens were cleaned by the acetone, ethanol, and distilled water for about 15 min with an ultrasonic cleaner, respectively.

### 2.3. Sample Characterization

The apparent contact angles and the contact angle hysteresis of the biological samples and the artificial superhydrophobic specimens were measured by a contact angle measuring instrument (OCA20, DATAPHYSICS, Stuttgart, Germany) at room temperature. In each measurement, 6 μL of deionized water was dispensed on the surface over a time span of 1.5 min. The measurement was averaged over at least six different locations for each.

A scanning electron microscope (SEM, ZEISS EVO 18, ZEISS, Oberkochen, Germany) and a laser confocal scanning microscope (LCSM, ZEISS OLS3000, ZEISS) were employed to obtain the surface morphology and structure of the samples. The chemical constituents of surfaces were detected by X-ray photoelectron spectroscopy (XPS, SPECS XR50, SPECS GmbH, Oberkochen, Germany).

As for measuring the contact time of the sample, two high-speed cameras (dimax.HS4, Pco, Germany and Phantom v5, VRI, Yorkshire, PA, USA), each filming at a minimum of 1500 frames/s, were used to capture the bouncing dynamic of water drop from the sequence of simultaneous top- and side-view images. The water drop (drop radius *R* = 1.5 mm) was released from a needle. The impact velocity was *V*_1_ = 1 m/s, which meant that the experiment was conducted in low speed condition. The needle was controlled by a system of syringe and syringe pump (115 VAC, Cole Parmer, Vernon Hills, IL, USA), which was used for controlling the impact velocity and the size of the drop. Thus, the dimensionless Weber number of each trial was identical according to the formula *We* = *ρV*_1_^2^*L*/*σ*, where *ρ*, *V*_1_, and *σ* are the density, the impact velocity, and the liquid–air surface tension, respectively. We used the initial radius of the drop (*R*) as the length scale (*L*). The Weber number of the droplet was kept at the same value in each trial by keeping the same initial velocity and the same size of the drop.

## 3. Results and Discussion

### 3.1. Wettability and Microstructure of Lotus Leaf and Cicada Wing

**Figure 1:**
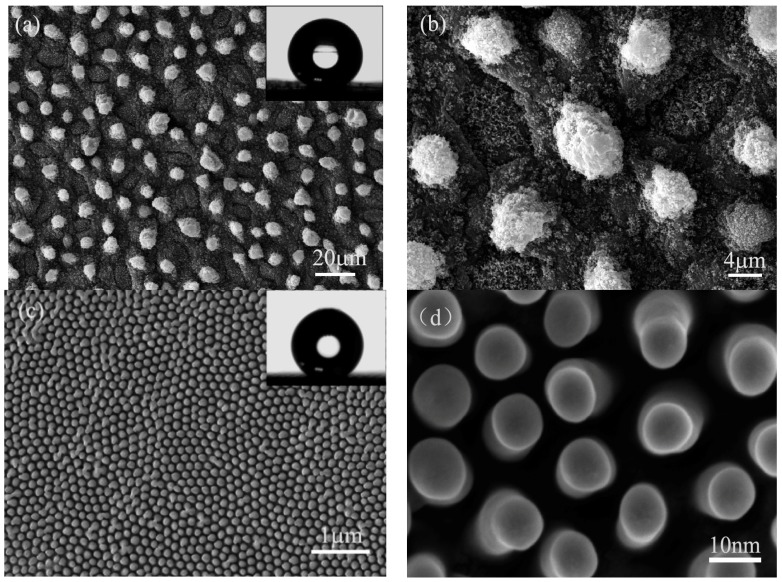
(**a**,**b**) Different magnifications of SEM images show microscopic structure of lotus leaf. Inset of (**a**): the apparent contact angle of lotus leaves is about 157° ± 1°; (**c**,**d**) Different magnifications of SEM images show microscopic structure of cicada wings. Inset of (**c**): the apparent contact angle of cicada wings is about 152° ± 1°.

The apparent contact angle of lotus leaves is about 157° ± 1° on the surface, which can be seen in the inset of Figure 1a. The contact angle hysteresis is about 3° on the lotus leaf surface. Different magnifications of SEM images (Figure 1a,b) show microscopic structure of lotus leaf. The array microstructure can be clearly observed, due to the uniform distribution of 5–15 mm scale papillae on the lotus surface shown in Figure 1a. A large amount of nanosticks (the average diameter is about 50 nm) are randomly distributed on the papillae, as shown in Figure 1b. Because of the synergic result of distribution of papillae array, covered with nanosticks and a wax layer on the surface, the lotus leaves exhibit superhydrophobicity and a self-cleaning property [6,28,29].

The apparent contact angle on the surface of cicada wings is about 152° ± 1°. However, even if the cicada wing surface was tilted at 90°, the water droplet did not slide from the surface. The cicada wings show superhydrophobic property having a high adhesive force with water, which is different from the lotus leaf. The mastoid structure with the diameter of 100 ± 20 nm, the spacing of 200 ± 20 nm, can be clearly seen on cicada wing surface (Figure 1c,d). It is interesting that the distribution of the mastoid structure conforms to the array microstructures as well.

### 3.2. Microstructure and Wettability of Artificial Superhydrophobic Surface 

**Figure 2:**
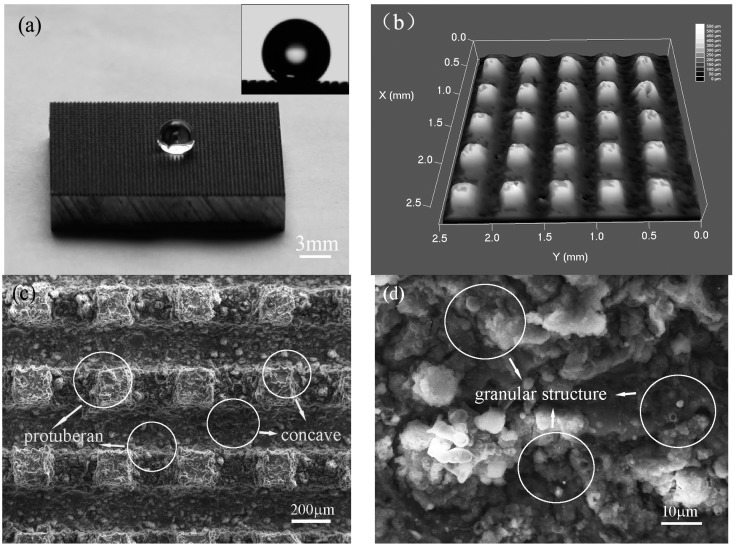
The photos and SEM images of the as-prepared aluminum alloy surface. (**a**) The photo of a water droplet on the as-prepared surface, and the inset of (**a**), the apparent contact angle of prepared aluminum alloy is 153° ± 1°; (**b**) The laser scanning confocal microscope image; (**c**,**d**) Different magnifications of SEM images on structured aluminum alloy surface after the HS-WEDM process.

Inspired by array microstructure existing in natural hydrophobic surfaces (lotus leaf and cicada wing), the array microstructure was transferred on a 7075 aluminum alloy substrate to form a superhydrophobic surface by using HS-WEDM without chemical modification. Figure 2 shows the photos and SEM image of the as-prepared aluminum alloy surface. A water droplet floats on the biomimetic superhydrophobic surface as shown in Figure 2a. In the inset of Figure 2a, the apparent contact angle of as-prepared surfaces is 154° ± 1°, and its contact angle hysteresis is less than 5°, showing a good superhydrophobic property. In addition, the three-dimensional surface image of the as-prepared surface obtained with the laser scanning confocal microscope is given in Figure 2b, which shows that the average height (*H*) of mastoid protrusion is about 200 μm. It can be clearly seen in Figure 2c that its mean length (*L*) and interval (*S*) are about 130 μm and 300 μm, respectively. Many micro-scale mastoid protrusions were an array configuration on the as-prepared surface. Moreover, the micro-scale mastoid protrusions are covered with countless randomly distributed micro-scale concaves and micro-scale protuberances generated during the manufacturing process (Figure 2c). A nano-scale granular structure can be seen on the biomimetic surfaces in Figure 2d. The 7075 aluminum alloy is a kind of hydrophilic materials of which intrinsic contact angle *θ_e_* is about 61°, which can be seen in the inset of Figure 6a. However, some changes took place following the machined array microstructure on the surface of the 7075 aluminum alloy. The wettability of the surface changes from hydrophilicity (the intrinsic contact angle is 61°) to superhydrophobicity (the contact angle is 153°) with a contact angle hysteresis less than 5°, and the advancing contact angle of the resulting surface reaches 154°. The wettability of the surface realized the transition from the hydrophilicity to the superhydrophobicity after the process of the array microstructure on the sample surfaces. In order to understand the superhydrophobic mechanism of the bionic surface, the X-ray photoelectron spectroscopy (XPS) experiment was performed to validate whether changes in chemical composition on the artificial surface, and simplified models of water droplets on the artificial surface were established to research the relationship between the wettability and the array microstructure of the artificial surface.

### 3.3. Superhydrophobic Mechanism of Artificial Surface

**Figure 3:**
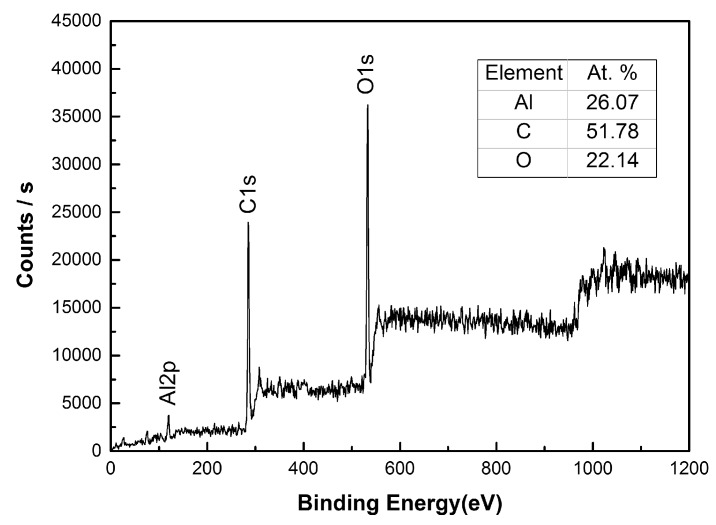
XPS spectrum of the artificial superhydrophobic surface.

The wettability of material surface is determined by the chemical composition and surface structure. The X-ray photoelectron spectroscopy (XPS) method was utilized to investigate the chemical composition of the artificial superhydrophobic surfaces. Figure 3 shows the XPS survey spectrum of the superhydrophobic surface. It reveals the presence of C, O, and Al on the surfaces. Strong peaks of C1s and O1s can be seen at 290.55 eV and 537.35 eV, respectively. Compared to the untreated aluminum alloy in Table 1, new elements (C and O) can be observed in XPS, and the atom proportions of carbon and oxygen increase to 51.78% and 22.14%, respectively. Analysis revealed that most of the mulch on the mastoid protrusion was oxides of aluminum. Moreover, these oxides formed micro-/nano-scale structures on the artificial surface, which made the Cassie state of artificial surface more stable.

**Figure 4:**
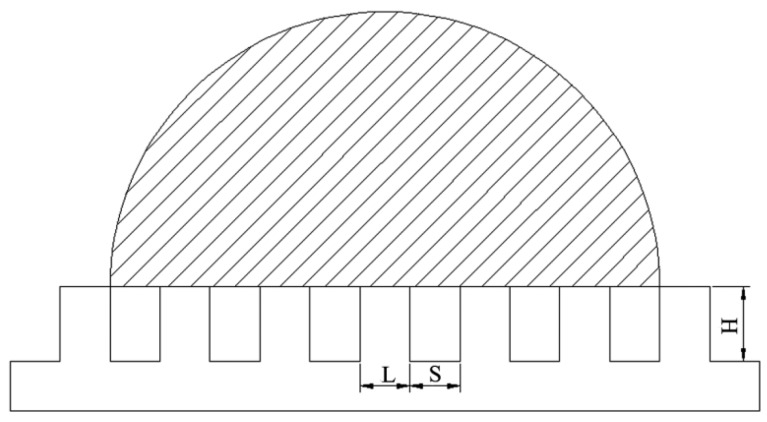
Regime of wetting according to the Cassie–Baxter model is depicted.

In order to study the relationship between the microstructure and wettability of the artificial surface, we simplified a model of water droplets on the artificial surface as shown in Figure 4. The model is a two-dimensional model diagrams of a water droplet on the artificial superhydrophobic surface, where *L*, *S*, and *H* represent side length, spacing, and height, respectively.

According to the “Cassie–Baxter” model [2,30,31], air can be trapped in the solid rough surface below a droplet, and the water drop is partially sitting on air (Figure 4). The trapped air could enhance surface hydrophobic property based on the Cassie–Baxter idea:

(1) $$\cos\theta_{w}=f\left(\cos\theta_{e}+1\right)-1$$

where *f* is the area fraction of the solid on the surface.

(2) $$f=\frac{L^{2}}{{\left(L+S\right)}^{2}}$$

Substituting (2) into (1), we have

(3) $$\cos\theta_{w}=\frac{L^{2}}{{\left(L+S\right)}^{2}}\left(\cos\theta_{e}+1\right)-1$$

Substituting *L* = 130, *S* = 300, *H* = 200 and *θ_e_* = 61° into Equation (3), the theoretical static contact angles of the “Cassie–Baxter” model was 149.8°. Results showed that the theoretically calculated value (149.8°) of the “Cassie–Baxter” model was close to the experimental value (153° ± 1°) measured by the contact angle measuring instrument. Conclusions can be drawn that the micro-scale mastoid protrusions form a bionic array microstructure, which is covered with micro-scale concaves and protuberances, and a nano-scale granular structure. The micro-/nano-scale structure is generated on the artificial surface, which can enhance the wettability of the artificial surface from the hydrophilicity to the superhydrophobicity by a trapped amount of air on the surface. In addition, new metal oxides and carbides generated in the machining process are another reason for the superhydrophobic on the artificial surface. 

### 3.4. Contact Time on the Artificial Surface and How to Reduce the Contact Time 

**Figure 5:**
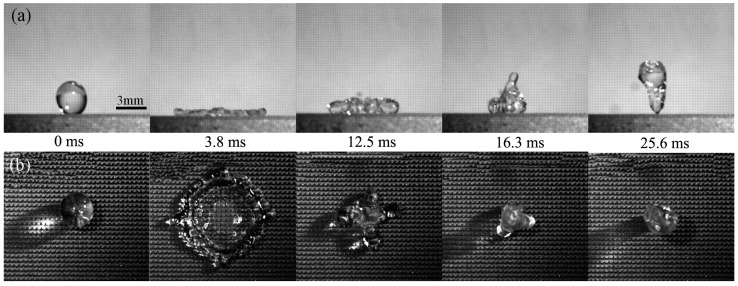
A water drop bouncing on the artificial surface (surface parameters: *L* = 130 μm, *S* = 300 μm, *H* = 200 μm). (**a**) Front-view images of a droplet bouncing on the artificial surface; (**b**) Synchronized top-view images of a droplet bouncing on this surface.

**Figure 6:**
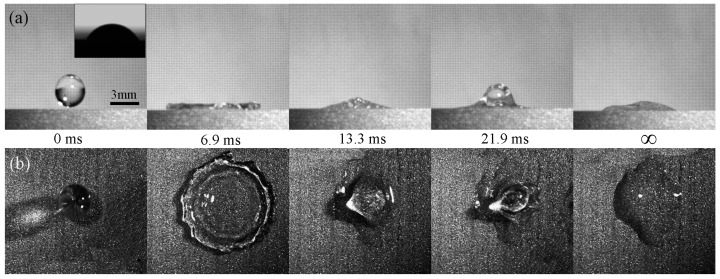
A water drop bouncing on the unprocessed 7075 surface. (**a**) Front-view images of a droplet bouncing on surface. Inset of (**a**): the contact angle of the surface is about 61°; (**b**) Synchronized top-view images of a droplet bouncing on the surface.

The contact time (*T_c_*: the time interval from the start of contacting the surface to lifting off) of the artificial surface was measured. The droplet (diameter *D* = 3 mm, impact *V*_1_ = 1.0 m/s) was used in the experiments and the bounce dynamics of the droplet were recorded by high-speed cameras from the front-view and the synchronized top-view. Figure 5 shows the droplet impacting process on the artificial surface. It can be clearly seen that the droplet spreads rapidly in the radial direction, reaches the maximum radius of the droplet, retracts, and then lifts off within 25.6 ms, which means that the contact time of the artificial superhydrophobic surface is 25.6 ms. The diameter of the droplet reaches the maximum value at 3.8 ms, and the droplet retracts to the largest extent at 12.5 ms and falls off the surface at 25.6 ms according to front-view images. The blank control group (unprocessed 7075 surface) was studied for comparison as shown in Figure 6. It can be clearly observed that the water droplet spreads and retracts but cannot bounce up. Therefore, the contact time of the water droplet on the unprocessed 7075 surface is regarded as infinite. Compared with the array microstructure surface, the time taken to reach the maximum diameter (*T_s_*) of the droplet on the unprocessed 7075 surface is 3.1 ms longer, and the time taken to retract the largest extent (*T_r_*) is longer as well. The experimental results indicate that the array structure possessing the micro-/nano-scale microstructure on the aluminum alloy surface not only improves the hydrophobic properties of the artificial surface, but also reduces the contact time, which could have a potential application in anti-icing. Different types of artificial surfaces (different array microstructures) have been designed and produced based on the previous work [29] in order to reduce the droplet contact time. An orthogonal experimental design has been achieved to analyze how the microstructure affects the contact time and to explore how contact time can be reduced.

The data of the surface parameters on different artificial superhydrophobic surfaces and the corresponding contact time and the apparent contact angle are shown in Table 2. An orthogonal array with nine test units was selected for the experiments. Obviously, different surface parameters correspond to different contact times. The analyzed results were shown in Table 3. In Table 3, *y_jk_* and *y*’*_jk_* are the apparent contact angle and the contact time corresponding to factor *j* and its level *k*, respectively. To assess the preferable level for factor *j*, the mean value *j_ky_* and *j*′*_ky_* are calculated, respectively. The value of the *R_j_* (*R_j_* = max[$\bar{y}$*_j_*_1_, $\bar{y}$*_j_*_2_, $\bar{y}$*_j_*_3_] − min[$\bar{y}$*_j_*_1_, $\bar{y}$*_j_*_2_, $\bar{y}$*_j_*_3_] or *R′_j_* = max[$\bar{y}$’*_j_*_1_, $\bar{y}$’*_j_*_2_, $\bar{y}$’*_j_*_3_] − min[$\bar{y}$*’_j_*_1_, $\bar{y}$’*_j_*_2_, $\bar{y}$’*_j_*_3_]) reflects the influence degree of the *j* factor (the greater the value of *R_j_*(*R′_j_*), the greater the influence degree of the *j* factor on the apparent contact angle or contact time). *L*(*L*′) row shows the preferable levels for each factor, and *S*(*S*′) row presents the most preferable set of these factors and levels. For the apparent contact angle, the preferable levels of the corresponding factors are indicated by the maximum mean value. However, for the contact time, the preferable levels of the corresponding factors are indicated by the minimum mean value. The combination of these preferable levels is the best combination of surface parameters in the experiment.

**Figure 7:**
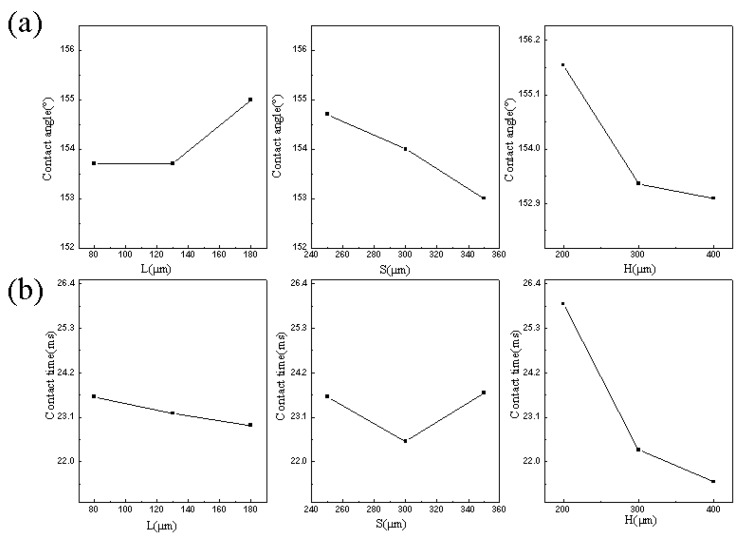
Tendency charts of (**a**) the apparent contact angle and (**b**) the contact time as a function of surface parameters.

Based on the orthogonal array with nine test units, Figure 7a,b show the effect of all factors on the apparent contact angle and the contact time, respectively. With the increase of *L* from 80 to 180 μm, the apparent contact angle remains unchanged until the *L* increasing to 130 μm and the contact time decreases with the increase of *L*. When *S* increased, the apparent contact angle slightly decreases and the contact time falls first and rises later. Both the apparent contact angle and the contact time decrease with the increase of *H*. The analyzed results, combining Table 3 and Figure 7, indicate that the order of importance about these factors is *H* > *S* > *L* for both the apparent contact angle and the contact time, which means that there is a close correlation between the apparent contact angle and the contact time, which owns a potential application in the surface processing and preparation in anti-icing and self-cleaning. 

The combination of *L*_3_*S*_1_*H*_1_ (*L*_3_ = 180 μm, *S*_1_ = 250 μm, *H*_1_ = 200 μm) is the preferable set of these factors and levels for the apparent contact angle, while the corresponding preferable set for the contact time is *L*_3_*S*_2_*H*_3_ (*L*_3_ = 180 μm, *S*_1_ = 300 μm, *H*_1_ = 400 μm), which has guiding significance in our future work to reducing the contact time by changing in surface parameters. 

## 4. Conclusions

In summary, inspired by the array microstructures of natural superhydrophobic surfaces (lotus leaf and cicada wing), the functional superhydrophobic surface on the 7075 aluminum alloy has been successfully fabricated by HS-WEDM without any chemical treatment. The wettability, the contact time on the artificial surface, and the decrease of the contact time have been studied in this research. The main conclusions are as follows:

- Lotus leaf and cicada wing have unique superhydrophobic properties. The apparent contact angle of lotus leaf and cicada wing is about 157° ± 1° and 151° ± 1°, respectively. The surfaces of the lotus leaf and cicada wing both possess an array microstructure, which is a very important factor for a natural superhydrophobic surface.
- Inspired by the array microstructure on the surfaces of the lotus leaf and cicada wing, the array microstructure had been successfully constructed by HS-WEDM on the surfaces of the 7075 aluminum alloy without any chemical treatment. The apparent contact angle of the artificial surface is about 153° ± 1°, with a contact angle hysteresis less than 5°, showing a good superhydrophobic property. During the machining process, surfaces of the aluminum alloy not only possess the array structure followed by micro-/nano-scale microstructure, but also generate new metal oxides and carbides. The fabrication of the superhydrophobic surface is simple, low-cost, and environmentally friendly.
- Different array microstructures have different effects on the wettability and contact time of the artificial superhydrophobic surface. The length (*L*), interval (*S*), and height (*H*) of the array microstructure are the main influential factors on the wettability and contact time. Changing these factors can control the wettability and the contact time. The order of importance of these factors is *H* > *S* > *L* for increasing the apparent contact angle and reducing the contact time, which would have a potential application in the surface processing and preparation in anti-icing and self-cleaning.

## Figures and Tables

**Table 1:** The chemical compositions of the 7075 aluminum alloy (wt %).

Element	Al (%)	Si (%)	Fe (%)	Cu (%)	Mn (%)	Mg (%)	Cr (%)	Zn (%)	Ti (%)
content	89.2	0.1	0.2	1.3	0.2	2.7	0.2	6	0.1

**Table 2:** The experimental surface parameters and results of the contact time and the apparent contact angle.

Sample No.	*L* Side Length of Protrusion (μm)	*S* Interval of Protrusion (μm)	*H* Height of Protrusion (μm)	*y_i_* APCA (°)	*y′_i_* Contact Time (ms)
1	(*L*_1_)80	(*S*_1_)250	(*H*_1_)200	155	25.3
2	(*L*_1_)80	(*S*_2_)300	(*H*_2_)300	153	22.7
3	(*L*_1_)80	(*S*_3_)350	(*H*_3_)400	152	22.8
4	(*L*_2_)130	(*S*_1_)250	(*H*_3_)400	154	22.7
5	(*L*_2_)130	(*S*_2_)300	(*H*_1_)200	153	25.6
6	(*L*_2_)130	(*S*_3_)350	(*H*_2_)300	151	21.4
7	(*L*_3_)180	(*S*_1_)250	(*H*_2_)300	156	22.8
8	(*L*_3_)180	(*S*_2_)300	(*H*_3_)400	153	19.1
9	(*L*_3_)180	(*S*_3_)350	(*H*_1_)200	156	26.8

**Table 3:** The analyzed results of the orthogonal array.

Indices	Item	*L*	*S*	*H*
APCA (°)	*y_j_*_1_	461	464	467
	*y_j_*_2_	461	462	460
	*y_j_*_3_	465	459	459
	$\bar{y}$*_j_*_1_	153.7	154.7	155.7
	$\bar{y}$*_j_*_2_	153.7	154	153.3
	$\bar{y}$*_j_*_3_	155	153	153
	*R_j_*	1.3	1.7	2.7
	*L*	*L*_3_	*S*_1_	*H*_1_
	*F*	*SHL*		
	*S*	*L*_3_*S*_1_*H*_1_		
Contact time (ms)	*y*’*_j_*_1_	70.8	70.8	77.7
	*y*’*_j_*_2_	69.7	67.4	66.9
	*y*’*_j_*_3_	68.7	71	64.6
	$\bar{y}$′*_j_*_1_	23.6	23.6	25.9
	$\bar{y}$’*_j_*_2_	23.2	22.5	22.3
	$\bar{y}$’*_j_*_3_	22.9	23.7	21.5
	*R*’*_j_*	0.7	1.2	4.4
	*L*’	*L*_3_	*S*_2_	*H*_3_
	*F*’	*HSL*		
	*S*’	*L*_3_*S*_2_*H*_3_		
